# Supplementary material for: Eye movements track prioritized auditory features in selective attention to natural speech
Source: Nat Commun. 2024 May 1;15:3692. doi: 10.1038/s41467-024-48126-2 (PMC11063150; doi:10.1038/s41467-024-48126-2)
Supplement: Supplementary file 1 — Supplementary Information [file 41467_2024_48126_MOESM1_ESM.pdf]

Supplementary Files

Supplementary Figures

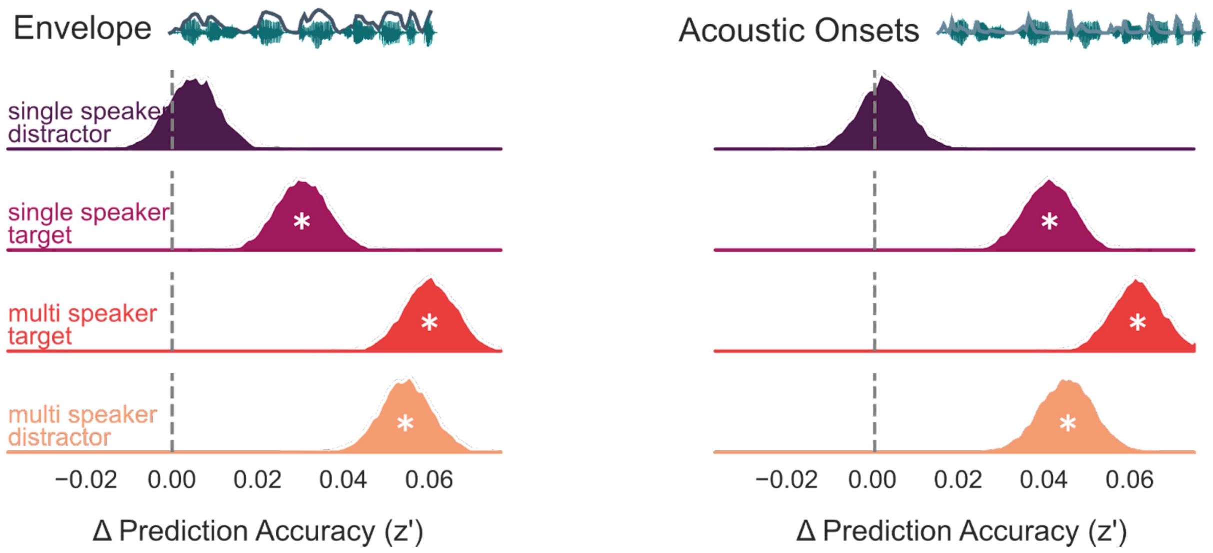

Fig. 1: **The effect of selective attention on speech tracking by horizontal eye movements. a** Differences in Fisher z-transformed prediction accuracies (**Δ** z') between models that additionally included the speech envelope and a control model for envelope (left panel) and acoustic onsets tracking (right panel) by horizontal eye movements. Statistics were performed using Bayesian regression models. A '*' within posterior distributions depicts a significant difference from zero (i.e. the 94%HDI does not include zero). *N* = 30.

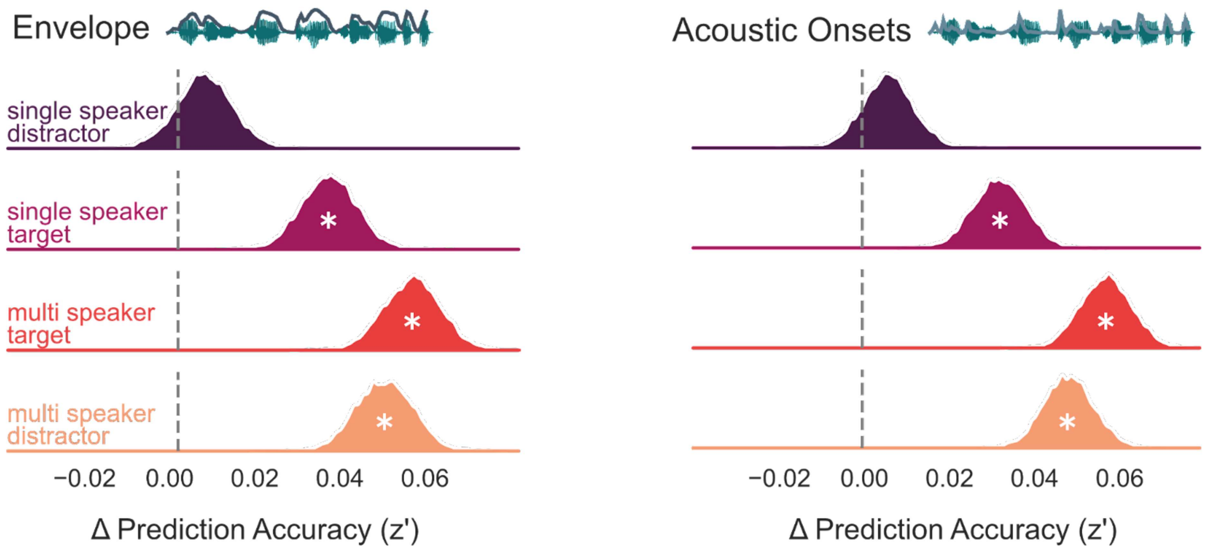

Fig. 2: **The effect of selective attention on speech tracking by vertical eye movements. a** Differences in Fisher z-transformed prediction accuracies (**Δ** z') between models that additionally included the speech envelope and a control model for envelope (left panel) and acoustic onsets tracking (right panel) by vertical eye movements. Statistics were performed using Bayesian regression models. A '*' within posterior distributions depicts a significant difference from zero (i.e. the 94%HDI does not include zero). *N* = 30.

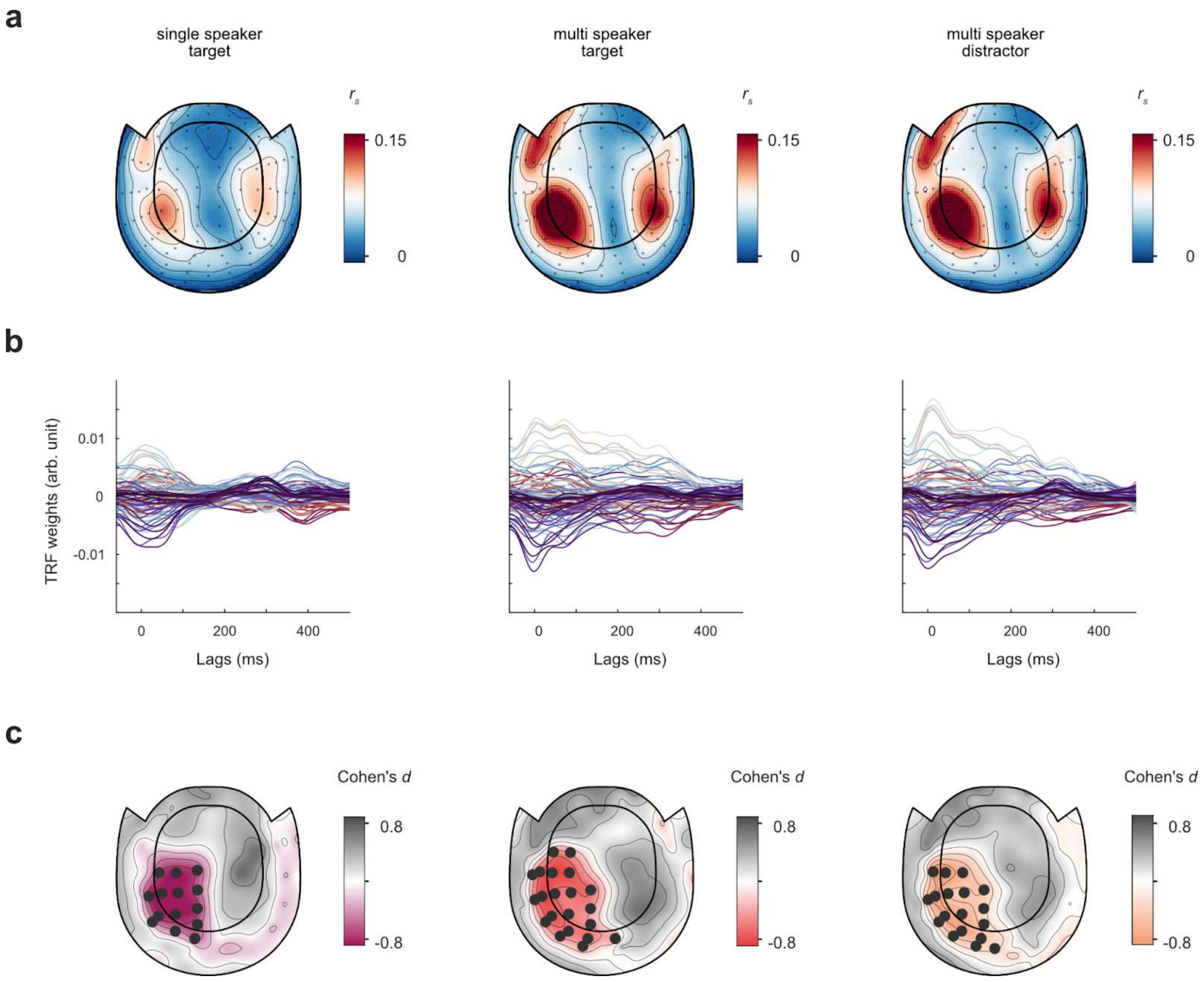

Fig. 3: **Controls for mediation analysis with boosting. a** Envelope encoding for 102 MEG magnetometers. Prediction accuracy is represented with spearman rho (r_s). **b** The corresponding envelope encoding weights. Both **a** and **b** show comparable patterns over sensors and time to the typically observed responses in neural speech tracking experiments. Note, however, that the single-trial design led to slightly weaker encoding in a single speaker condition compared to a multi speaker condition as well as early-latency responses ~ 0 ms due to the predictable 5-word sentence structure. **c** The mediation effect of neural speech tracking via shuffled eye-movements. In order to ensure that the mediation effect did not simply arise from adding an additional feature (i.e. eye movements) to the boosting model, we repeated the same analysis with a shuffled version of eye movements (over time within conditions). Eye movements shared contributions to neural speech processing mostly over left parietal sensors for the target in a single speaker condition ($t(29) = -4.10$, $p < 0.001$, Cohen's d = -0.74), the target in a multi speaker condition ($t(29) = -3.58$, $p < 0.001$, Cohen's d = -0.65) as well as the distractor in a multi speaker condition ($t(29) = -3.88$, $p < 0.001$, Cohen's d = -0.71. This confirmed the result presented within the main article and ruled out the potential bias due to feature dimensions. $N = 30$.

Supplementary Tables

Supplementary Table 1

Model summary statistics for encoding of acoustic features (Fisher z-transformed ranks) depending on condition

	speech envelope				acoustic onsets			
	b	*sd*	hdi 3%	hdi 97%	*b*	*sd*	hdi 3%	hdi 97%
single speaker - distractor	0.00566	0.00487	-0.00351	0.01474	0.00443	0.00546	-0.00566	0.01504
single speaker - target	0.03175	0.00479	0.02242	0.04055	0.04031	0.00536	0.03002	0.05029
multi speaker - target	0.05963	0.00500	0.05058	0.06924	0.05984	0.00564	0.04917	0.07021
multi speaker - distractor	0.05301	0.00488	0.04358	0.06184	0.04822	0.00559	0.03751	0.05834

Note: Dependent Variable = encoding results: encoding model - control model (average over channels)

Supplementary Table 2

Model summary statistics for intelligibility depending on encoding of acoustic features

	speech envelope				acoustic onsets			
	b	*sd*	hdi 3%	hdi 97%	*b*	*sd*	hdi 3%	hdi 97%
Intercept (single speaker)	4.71761	0.14062	4.45883	4.98430	4.71904	0.15180	4.43055	4.99684
Condition (multi speaker)	-2.33610	0.16900	-2.64686	-2.01496	-2.33834	0.17396	-2.67133	-2.02747
Encoding	19.11251	5.50027	8.85920	29.2232	11.69498	5.96981	0.57023	23.10985
Encoding (multi speaker) x Condition	-13.22355	6.61445	-25.21237	-0.58644	-5.38008	6.43128	-17.82181	6.20978

Note: Independent Variable = encoding results: encoding model - control model (average over channels)

Supplementary Table 3

Model summary statistics for subjective effort depending on encoding of acoustic features

	speech envelope				acoustic onsets			
	b	*sd*	hdi 3%	hdi 97%	*b*	*sd*	hdi 3%	hdi 97%
Intercept (single speaker)	2.31823	0.16192	1.99954	2.61297	2.32003	0.15949	2.00677	2.61029
Condition (multi speaker)	1.71252	0.18781	1.36073	2.07069	1.70273	0.18035	1.35386	2.03397
Encoding	-8.04769	6.08016	-19.25225	3.66282	-4.22670	6.23876	-15.64272	7.83369
Encoding (multi speaker) x Condition	2.34972	7.34763	-11.44273	15.96361	-4.88605	6.55999	-17.31028	7.41695

Note: Independent Variable = encoding results: encoding model - control model (average over channels)

Supplementary Methods

To investigate whether subjective ratings of listening effort and task engagement differed between conditions, we calculated two additional models including only the attended speech conditions (multi vs. single speaker):

$$\text{effort} \sim \text{condition} + (1|\text{subject})$$

$$\text{engagement} \sim \text{condition} + (1|\text{subject})$$

Listening effort was rated higher in the multispeaker condition compared to the single speaker condition (*b* = 1.709, 94%HDI = [1.350, 2.074]). However, we found no difference in task engagement between the two conditions (*b* = 0.085, 94%HDI = [-0.122, 0.294]).

In order to investigate the relationship between intelligibility and ocular speech tracking (Conditions 2 & 3) in a more robust version that excludes potential behavioral outliers, we inspected the behavioral data for 'outliers' based on 1.5*inter quartile range and found two participants (id 7 and 18) to be present in both conditions (note, however, their effort score as well as the very high intelligibility scores of 94% and 96% for 'outliers' in the single speaker condition, and still 80% intelligibility for subject_id 7 in the multi speaker condition):

Single speaker

Id	Intelligibility (effort)
2	0.967347 (4)
7	0.943000 (3.5)
18	0.922667 (2.5)

Multi speaker

Id	intelligibility

4	0.747000 (4.5)
7	0.806250 (4.5)
18	0.653061 (5)
23	0.838583 (5)

We then reran the modeling for intelligibility based on the dataset that excludes these two participants, again using the updated formula from the previous revision where intelligibility was logit-transformed and envelope tracking represented in fisher-z space:

intelligibility ~ condition * envelope tracking + (1|subject)

Importantly, we still observed a positive effect for the encoding of the speech envelope (β = 13.40829, 94%HDI = [2.47948, 23.40420]) as in the main analysis. However, we no longer find evidence for an interaction effect (β = -11.51946, 94%HDI = [-25.20734, 0.88333]).
