# Supplementary material for: Eye movements track prioritized auditory features in selective attention to natural speech
Source: Nat Commun. 2024 May 1;15:3692. doi: 10.1038/s41467-024-48126-2 (PMC11063150; doi:10.1038/s41467-024-48126-2)
Supplement: Supplementary file 3 — Reporting Summary [file 41467_2024_48126_MOESM3_ESM.pdf]

nature portfolio

Corresponding author(s): Quirin Gehmacher

Last updated by author(s): Feb 7, 2024

Reporting Summary

Nature Portfolio wishes to improve the reproducibility of the work that we publish. This form provides structure for consistency and transparency in reporting. For further information on Nature Portfolio policies, see our Editorial Policies and the Editorial Policy Checklist.

Statistics

For all statistical analyses, confirm that the following items are present in the figure legend, table legend, main text, or Methods section.

n/a	Confirmed	
☐	☒	The exact sample size (*n*) for each experimental group/condition, given as a discrete number and unit of measurement
☐	☒	A statement on whether measurements were taken from distinct samples or whether the same sample was measured repeatedly
☐	☒	The statistical test(s) used AND whether they are one- or two-sided<br>*Only common tests should be described solely by name; describe more complex techniques in the Methods section.*
☐	☒	A description of all covariates tested
☐	☒	A description of any assumptions or corrections, such as tests of normality and adjustment for multiple comparisons
☐	☒	A full description of the statistical parameters including central tendency (e.g. means) or other basic estimates (e.g. regression coefficient) AND variation (e.g. standard deviation) or associated estimates of uncertainty (e.g. confidence intervals)
☐	☒	For null hypothesis testing, the test statistic (e.g. *F*, *t*, *r*) with confidence intervals, effect sizes, degrees of freedom and *P* value noted<br>*Give P values as exact values whenever suitable.*
☐	☒	For Bayesian analysis, information on the choice of priors and Markov chain Monte Carlo settings
☒	☐	For hierarchical and complex designs, identification of the appropriate level for tests and full reporting of outcomes
☐	☒	Estimates of effect sizes (e.g. Cohen's *d*, Pearson's *r*), indicating how they were calculated

Our web collection on statistics for biologists contains articles on many of the points above.

Software and code

Policy information about availability of computer code

Data collection	The experiment was coded and conducted with Psychtoolbox-3 (D. H. Brainard & Vision, 1997; Kleiner et al., 2007) implemented in Matlab R2020b (The MathWorks, Natick, Massachusetts, USA) with an additional class-based library (‘Objective Psychophysics Toolbox’, o_ptb; Hartmann & Weisz, 2020). As of now, experimental code is not publicly available, but can of course be made available as open source.
Data analysis	Code to analyze preprocessed data and further reproduce results and figures from this manuscript is available at the corresponding author’s GitLab repository (https://gitlab.com/qubitron/ocularspeechtracking). In summary, for data analysis , we used Matlab R2020b with the FieldTrip Toolbox (Oostenveld et al., 2011), and the open-source mTRF-Toolbox (Crosse et al., 2016, 2021). For statistical analysis we used Bayesian multilevel regression models with Bambi (Capretto et al., 2020) , a python package built on top of the PyMC3 package (Salvatier et al., 2016) , for probabilistic programming.

For manuscripts utilizing custom algorithms or software that are central to the research but not yet described in published literature, software must be made available to editors and reviewers. We strongly encourage code deposition in a community repository (e.g. GitHub). See the Nature Portfolio guidelines for submitting code & software for further information.

Data

Policy information about availability of data

All manuscripts must include a data availability statement. This statement should provide the following information, where applicable:

- Accession codes, unique identifiers, or web links for publicly available datasets
- A description of any restrictions on data availability
- For clinical datasets or third party data, please ensure that the statement adheres to our policy

Preprocessed Data required to reproduce the analyses supporting this work are publicly available in the Open Science Framework repository (https://osf.io/m6rfq). The raw data are protected and are not publicly available due to data privacy laws but can be made available upon reasonable request. Source data are provided with this paper.

Research involving human participants, their data, or biological material

Policy information about studies with human participants or human data. See also policy information about sex, gender (identity/presentation), and sexual orientation and race, ethnicity and racism.

Reporting on sex and gender	Findings apply to all sex and gender. Participants gave written self-reports on their sex and gender along with informed consent. We did not aim for and therefore not perform sex and gender based analysis as our sample size of 30 participants would not allow for exhaustive power within groups. However, we aimed to keep numbers of males and females, as stated within their written self-reports on sex, roughly equal. We ended up with 19 females and 11 males as stated by participants' sex.
Reporting on race, ethnicity, or other socially relevant groupings	People were not classified into differnt groups in this study
Population characteristics	30 healthy participants (19 female, M age = 26.27, SD age = 9.08) were recruited. All participants were German native speakers, reported normal hearing, and (corrected to) normal vision. Participants gave written, informed consent and reported no previous neurological or psychiatric disorders.
Recruitment	Participants were recruited using the University of Salzburg's SONA-System. The study was advertised on this portal, giving a rough descrition of the study (where, when, what will be investigated, i.e. listening in challenging situations). Eligibility requirements for participation were the following:<br>Age of 18-65, no ferromagnetic metal in the body that cannot be temporarily removed (due to MEG measurements), german as native language as the study contained speech stimuli in german. It has to be shown that the effects presented in the study are consistend across languages, however, many studies show comparable effects for speech processing across languages with e.g. syllable rate.<br>The sample might be biased by a high proportion of university students due to the SONA-systems advertisement. Participants were deciding on their participation instead of random selection, hence potentially special interest in the topic could have a bias on the study due to self-selection.
Ethics oversight	The study protocol was approved by the ethics committee of the University of Salzburg and was carried out in accordance with the declaration of Helsinki

Note that full information on the approval of the study protocol must also be provided in the manuscript.

Field-specific reporting

Please select the one below that is the best fit for your research. If you are not sure, read the appropriate sections before making your selection.

☒ Life sciences ☐ Behavioural & social sciences ☐ Ecological, evolutionary & environmental sciences

For a reference copy of the document with all sections, see nature.com/documents/nr-reporting-summary-flat.pdf

Life sciences study design

All studies must disclose on these points even when the disclosure is negative.

Sample size	No sample size calculation was performed prior to the experiment. We aimed for a sample size of 30 participants as it is the standard of our lab for repeated measurement studies (e.g. Demarchi, Sanchez, and Weisz 2019, Schubert et al. 2023, Schmidt et al., 2020) and in general on the upper end for comparable MEG studies. In addition, the simultaneous Eye-Tracking recording was time consuming due to additional calibration / validation procedures which was another limiting factor. No further specifications were made with regards to sample selection. Data collection was stopped once the target sample size was reached and prior booked MEG measurement time slots were used up.<br><br>Demarchi, G., Sanchez, G., & Weisz, N. (2019). Automatic and feature-specific prediction-related neural activity in the human auditory system. Nature communications, 10(1), 3440.<br>Schubert, J., Schmidt, F., Gehmacher, Q., Bresgen, A., & Weisz, N. (2023). Cortical speech tracking is related to individual prediction tendencies. Cerebral Cortex, 33(11), 6608-6619.

Schmidt, F., Demarchi, G., Geyer, F., & Weisz, N. (2020). A backward encoding approach to recover subcortical auditory activity. NeuroImage, 218, 116961.

Data exclusions	On rare events, the synchronizatin of the Eye-Tracker with the MEG-System led to buffer issues which caused a salient noise during speech stimulation of one or two trials for a few participants. We therefore excluded those trials from later analysis and randomly subsampled the same amount of trials for all other participants. In sum, 98 trials per condition (of 100) were retained for further analysis.
Replication	In order to ensure a high amount of reproducibility / replicability, we used a highly standardized and well tested stimulus material (OLSA Sentences), minimal preprocessing steps, cross-validation with a regulariztion parameter across all subjets & conditions, robust Bayesian satistics with t-distributions, and made preprocessed data and code publicly available (see above)
Randomization	The repeated measures task consisted of three conditions split into six blocks of 50 trials, i.e. 100 trials per condition. The order of the blocks and trials was randomized across participants.
Blinding	Blinding was not necessary to this study as there were no group allocations. All participants were performing a repeated measures design where conditions and blocks were randomized (see above).

Reporting for specific materials, systems and methods

We require information from authors about some types of materials, experimental systems and methods used in many studies. Here, indicate whether each material, system or method listed is relevant to your study. If you are not sure if a list item applies to your research, read the appropriate section before selecting a response.

Materials & experimental systems

n/a	Involved in the study
☒	☐ Antibodies
☒	☐ Eukaryotic cell lines
☒	☐ Palaeontology and archaeology
☒	☐ Animals and other organisms
☒	☐ Clinical data
☒	☐ Dual use research of concern
☒	☐ Plants

Methods

n/a	Involved in the study
☒	☐ ChIP-seq
☒	☐ Flow cytometry
☒	☐ MRI-based neuroimaging
